# Ultrafast Laser Pulse Induced Transient Ferrimagnetic State and Spin Relaxation Dynamics in Two-Dimensional Antiferromagnets

**DOI:** 10.1021/acs.nanolett.3c02727

**Published:** 2023-08-29

**Authors:** Junjie He, Shuo Li, Thomas Frauenheim, Zhaobo Zhou

**Affiliations:** † Faculty of Science, Department of Physical and Macromolecular Chemistry, Charles University, Prague 12843, Czech Republic; ‡ Institute of Advanced Study, Chengdu University, Chengdu 610100, China; § School of Science, Constructor University, Bremen 28759, Germany; ∥ Bremen Center for Computational Materials Science, University of Bremen, Bremen 28359, Germany

**Keywords:** 2D magnetism, spin dynamics, spin relaxation, real-time TDDFT, nonadiabatic MD, antiferromagnetism

## Abstract

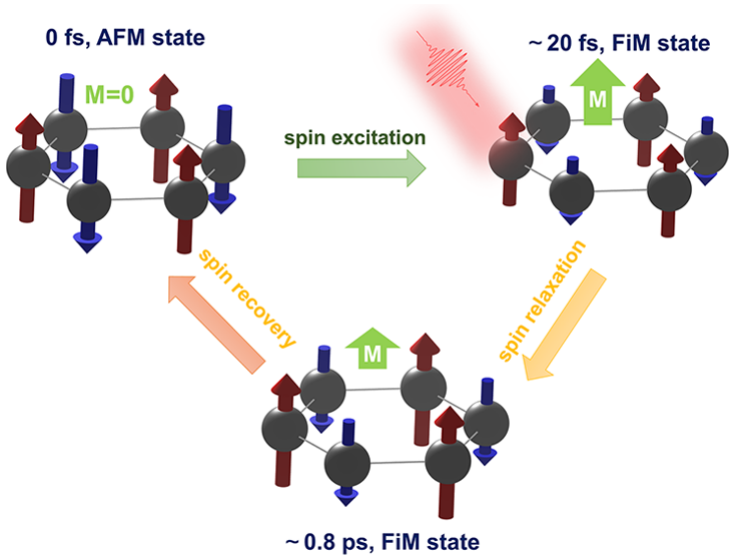

We employ real-time time-dependent density functional theory (rt-TDDFT) and ab initio nonadiabatic molecular dynamics (NAMD) to systematically investigate the ultrafast laser pulses induced spin transfer and relaxation dynamics of two-dimensional (2D) antiferromagnetic-ferromagnetic (AFM/FM) MnPS_3_/MnSe_2_ van der Waals heterostructures. We demonstrate that laser pulses can induce a ferrimagnetic (FiM) state in the AFM MnPS_3_ layer within tens of femtoseconds and maintain it for subpicosecond time scale before reverting to the AFM state. We identify the mechanism in which the asymmetric optical intersite spin transfer (OISTR) effect occurring within the sublattices of the AFM and FM layers drives the interlayer spin-selective charge transfer, leading to the transition from AFM to FiM state. Furthermore, the unequal electron–phonon coupling of spin-up and spin-down channels of AFM spin sublattice causes an inequivalent spin relaxation, in turn extending the time scale of the FiM state. These findings are essential for designing novel optical-driven ultrafast 2D magnetic switches.

Ultrafast laser pulse induced magnetization dynamics provide unprecedented opportunities for the manipulation and control of the spin of magnetic materials with extremely high speed, reaching time scales of a few femtoseconds or even attoseconds.^1^ This remarkable capability holds great potential for a wide range of technological applications, including magnetic data storage and ultrafast spintronics. Extensive research efforts have been devoted to investigating the ultrafast control of magnetism and understanding the mechanism of the angular momentum transfer in itinerant ferromagnetic (FM) metals, including Ni, Fe, and so on.^1^ Antiferromagnetic (AFM) materials, characterized by opposing spin alignments and the absence of macroscopic magnetization, offer numerous advantages such as faster dynamics, improved stability, and reduced stray fields.^2,3^ However, the lack of macroscopic magnetization in AFM makes it challenging to experimentally detect ultrafast magnetization dynamics. For instance, Thielemann-Kühn et al.^4^ showed that optical manipulation of AFM order is considerably faster than that of FM, arising that this angular momentum transfer channel is effective in magnetic metals with antiparallel spin alignment, which provides a possibility that reversible changes in the magnetization state of antiferromagnets. Such a magnetic state transition has been observed in the time domain for the first time in FeRh on a subpicosecond time scale after excitation with femtosecond laser pulses.^5,6^ Very recently, Golias et al.^7^ reported on the ultrafast optically induced FM alignment of AFM Mn in Co/Mn multilayers. However, the physical mechanism of ultrafast spin transfer dynamics in AFM materials is still not fully understood.

Two-dimensional (2D) magnetism has garnered significant interest due to the extraordinary features exhibited by a diverse range of intrinsic 2D magnetic van der Waals crystals, including CrI_3_, CrGeTe_3_, FePS_3_, NiPS_3_, MnPS_3_, and Fe_3_GeTe_2_, among others.^8−13^ These materials have demonstrated exceptional optical, magnetic, magneto-electric, and magneto-optic properties, presenting exciting opportunities to manipulate spin and magnetism in the 2D limit.^14^ Furthermore, 2D magnets can be integrated into van der Waals (vdW) heterostructures by stacking them with conventional 2D materials or other 2D magnets, creating a plethora of vdW heterostructures with unique properties. The proximity effect in these heterostructures can give rise to intriguing quantum properties, such as the quantum anomalous Hall effect and Valley pseudospin, providing a rich playground for exploring novel quantum phenomena.^15,16^ Recent advancements in the field have unveiled the captivating emergence of light control over spin dynamics, all-optical switching, and valley polarization properties in 2D magnets and their heterostructures.^17−21^ Notably, Seyler et al.^22^ demonstrated that photoexcitation can manipulate interlayer spin transfer and proximity effects, allowing for the tuning of valley polarization in CrI_3_/MoSe_2_ heterostructures. Additionally, Zhang et al.^17^ have found circularly polarized light pulses inducing all-optical magnetization switching in the atomically thin FM CrI_3_ semiconductor. These findings highlight the tremendous potential of light excitation as a tool for manipulating and controlling magnetism in 2D magnets. However, despite these significant advances, the underlying physical mechanism of laser-pulse-induced spin dynamics in 2D magnetic systems remains poorly explored.

The laser-induced spin dynamics have been successfully described by full quantum mechanical theory and real-time time-dependent density-functional theory (rt-TDDFT). Dewhurst et al.^23,24^ have theoretically proposed the optical intersite spin transfer (OISTR) effect, that the optical excitation can directly, coherently, and efficiently redistribute spins between different magnetic sublattices in multicomponent magnetic materials. Several experimental results have also confirmed the OISTR effect in various magnetic systems, including magnetic multilayer and Heusler compounds.^25−29^ Nonetheless, rt-TDDFT can now describe spin interaction only in the pure electronic system, which limits the applicable simulation time to the first ∼100 fs. Coupling spin dynamics with additional nuclei degrees of freedom to a longer time scale remains a challenge.

In this work, we describe the laser pulse induced spin transfer and relaxation dynamics processes in AFM/FM MnPS_3_/MnSe_2_ heterostructures based on state-of-the-art rt-TDDFT and ab initio nonadiabatic molecular dynamics (NAMD) simulation. Our rt-TDDFT results demonstrate that the ultrafast laser pulses can induce spin-selective charge transfer in the MnPS_3_/MnSe_2_ heterostructure, generating a ferrimagnetic (FiM) state with a net magnetic moment of 1 μ_B_ in the AFM MnPS_3_ layer within tens of femtoseconds. Such an FiM state can be maintained on a subpicosecond time scale after the laser pulse disappears and then reverts to the AFM state, based on NAMD simulations. The microscopic mechanism behind magnetic state transition and FiM state relaxation originates from the asymmetrical interlayer spin transfer and relaxation process.

## Structural, Electronic, and Magnetic Properties

**Figure 1:**
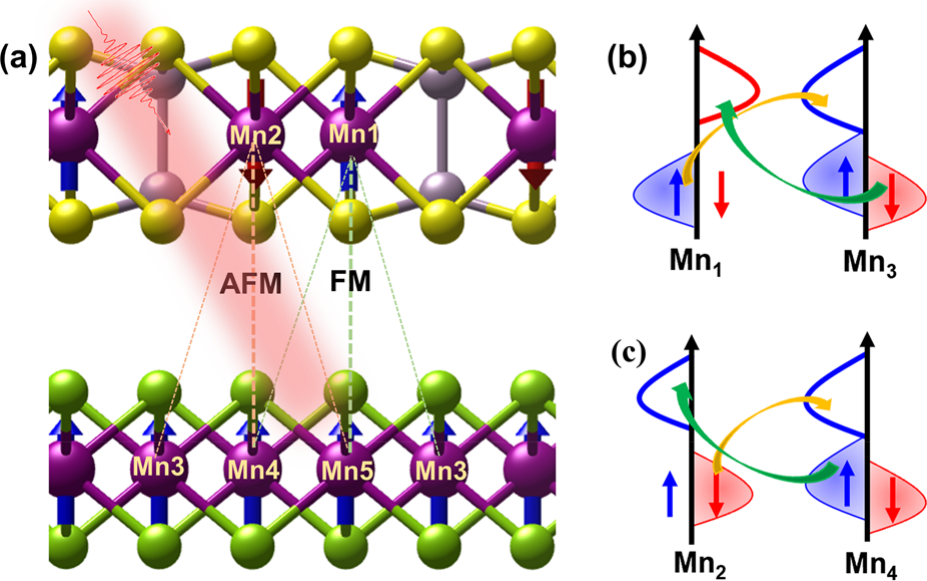
The vdW heterostructure model and spin transfer pathway for AFM and FM. Side view of (a) MnPS_3_/MnSe_2_ heterostructure with the coexistence of interlayer AFM and FM magnetic order. Schematic overview for OISTR effect induced interlayer (b) AFM (between Mn1 to Mn3) and (c) FM (between Mn2 to Mn4) spin transfer.

We constructed a vdW heterostructure model depicted in Figure 1, which consists of an AFM semiconductor layer, MnPS_3_, and a metallic FM layer, MnSe_2_. MnPS_3_ exhibits a honeycomb spin–lattice, while MnSe_2_ has a triangular spin–lattice. Our density functional theory (DFT) calculations confirm in-plane lattice parameters of 6.09 and 3.47 Å for MnPS_3_ and MnSe_2_, respectively, consistent with recent works.^30,31^ To achieve a proper match, we construct a 2 × 2 supercell of MnSe_2_ that aligns well with a 1 × 1 supercell of MnPS_3_. Using DFT calculations, we optimize the atomic structures by incorporating van der Waals interactions within the generalized gradient approximation (GGA) and the Hubbard U. We also consider various stacking configurations for the MnPS_3_/MnSe_2_ vdW heterostructure. We selected the most stable stacking configuration, illustrated in Figure 1a, for subsequent investigation of the spin dynamics.

At the interface between the MnPS_3_/MnSe_2_ heterostructure, the MnPS_3_ layer exhibits in-plane AFM order. The interlayer exchange interaction between MnPS_3_ and MnSe_2_ results in the alignment of the spins of the MnPS_3_ layer either parallel or antiparallel to the FM MnSe_2_ spins. As depicted in Figure 1a, the Mn1 and Mn2 atoms in the MnPS_3_ layer exhibit FM and AFM coupling to the Mn atoms in the MnSe_2_ layer, respectively. This coexistence of AFM and FM interlayer exchange interactions gives rise to distinct interlayer spin transfer. Additionally, we calculated the atom-resolved projected band structures for Mn1, Mn2, and Mn3 atoms (see Figure S1). The results reveal that the majority states of Mn1 in the MnPS_3_ layer are distributed around the occupied energy levels of −1.2 eV and in the energy range of [-3, −4] eV, while the minority orbitals located in the empty states within the energy window of [1, 3] eV. Mn2 atoms of the AFM layer exhibit an identical orbital distribution to Mn1 but with opposite spin channels due to their AFM coupling. As for Mn3 atoms in the FM layer, both majority and minority states are relatively delocalized, reflecting the low spin state of Mn3 atoms. Consequently, the antiparallel alignment of Mn1/Mn2 and the parallel alignment of Mn3/Mn4 enable efficient spin-selective charge transfer upon photoexcitation, as illustrated in Figure 1b and c. This phenomenon likely leads to intriguing interlayer spin transfer dynamics of AFM/FM heterostructures and magnetic state transitions in the top MnPS_3_ layer.

## Laser Pulse Induced Interlayer Spin Transfer Dynamics

**Figure 2:**
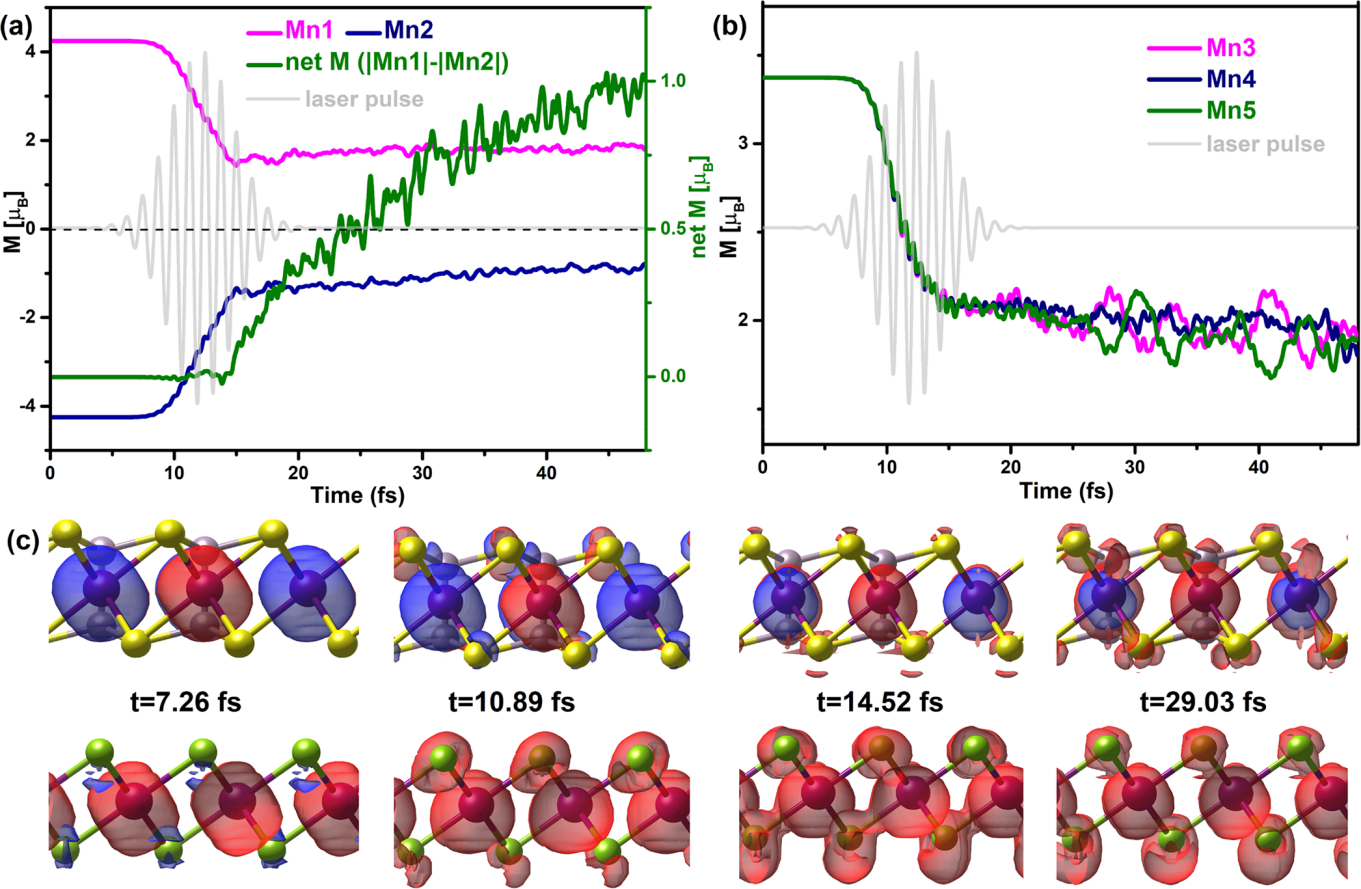
Time-dependent spin dynamics and spin density. (a) Time-dependent dynamics of the local magnetic moment for Mn1 (pink) and Mn2 (blue), and the difference between Mn1 and Mn2 (green) in the AFM MnPS_3_ layer. (b) Time evolution of the local magnetic moment for the FM MnSe_2_ layer. The vector potential **A**(*t*) of the laser pulse is also shown. (c) Snapshots of the spin density at *t* = 7.26 fs, *t* = 10.89 fs, *t* = 14.52 fs, and *t* = 29.03 fs. The iso-surface is set to 0.005 e/Å^3^. Red and blue represent spin up and down density, respectively.

Next, we investigate the spin transfer dynamics occurring at the van der Waals interface of the AFM/FM MnPS_3_/MnSe_2_ heterostructure. To gain insights into this process, we perform rt-TDDFT calculations using the full potential augmented plane-wave ELK code (for detailed computational information, refer to the Supporting Materials). To initiate the dynamics, a linearly polarized ultrashort pulse laser with in-plane polarization is applied to the MnPS_3_/MnSe_2_ heterostructure. The laser pulse has a photon energy of 3.2 eV, a full width at half-maximum (fwhm) of 6.04 fs, and a fluence of 24.6 mJ/cm^2^. The vector potential **A**(*t*) of the laser pulse is depicted in Figure 2a and b. The evolution of element-resolved spin dynamics is depicted in Figure 2a. Both Mn1 and Mn2 atoms exhibit significant demagnetization upon the arrival of the pump pulse. Notably, there is an intriguing asymmetry in the demagnetization behavior of Mn1 and Mn2 atoms as the pump pulse reaches its peak and half-maximum at approximately 15 fs. Starting from this point, the Mn1 and Mn2 atoms undergo uncompensated magnetic moment changes. Consequently, the net magnetism of the AFM MnPS_3_ gradually increases throughout the simulation duration (50 fs), indicating the emergence of an induced FiM state in the AFM MnPS_3_. Conversely, in the FM MnSe_2_ layer, Mn3, Mn4, and Mn5 atoms exhibit a pronounced demagnetization upon reaching the peak of the pump pulse, followed by saturation of diamagnetization as the pulse reaches its peak and concludes. Subsequently, even after the laser pulse, a slow demagnetization of Mn3, Mn4, and Mn5 atoms persists, corresponding to the enhancement of the net magnetic moment of the AFM layer. To further visualize the laser-induced FiM state in the AFM MnPS_3_, the time-dependent spin density dynamics are displayed in Figure 2c, which clearly illustrate the demagnetization in the heterostructures and the uncompensated magnetic moments of Mn1 and Mn2 atoms within the AFM layer.

**Figure 3:**
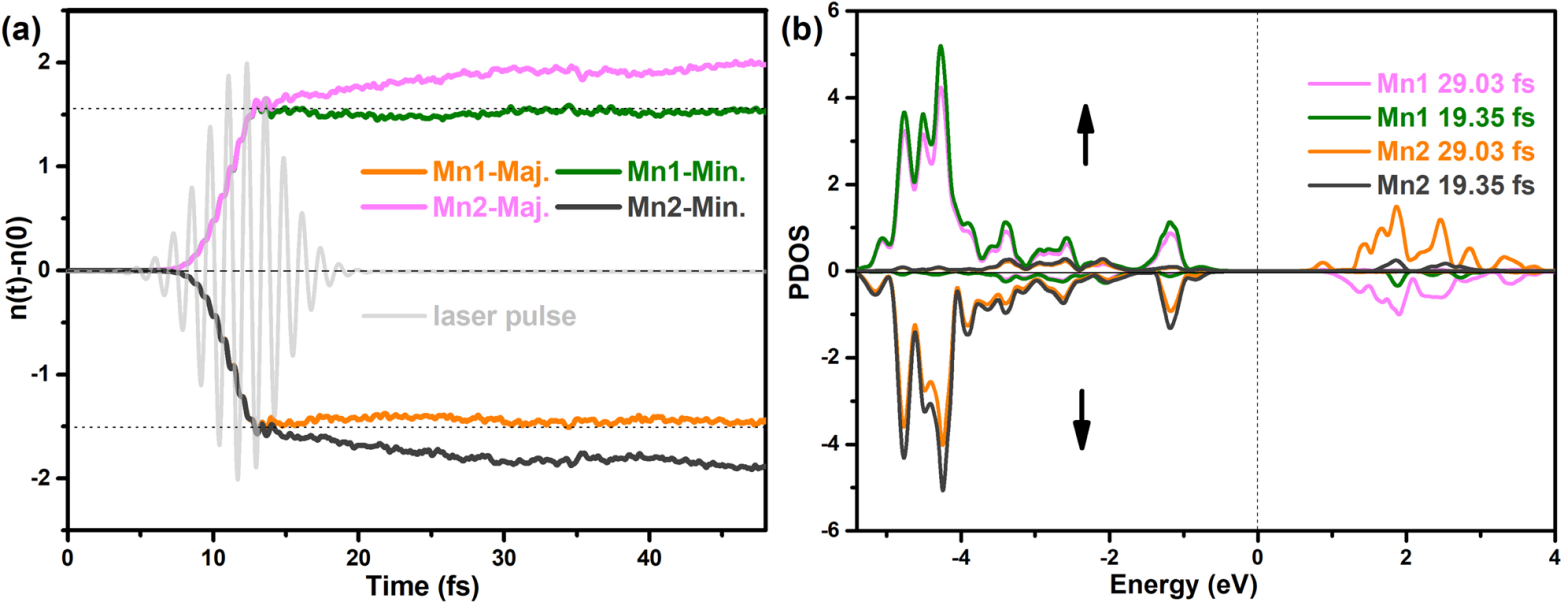
Time-dependent occupation dynamics and projected density of states (PDOS). (a) Time-dependent change of majority (Maj.) and minority (Min.) occupations as a function of time (in fs) of Mn1 and Mn2 atoms, which is defined as *n*(*t*) – *n*(0); here, the gray line represents the vector potential **A**(*t*) of the laser pulse. (b) Time-dependent PDOS of Mn1 and Mn2 atom for the MnPS_3_ monolayer. The upward and downward arrows represent the spin up and down, respectively.

Next, we will analyze the physical mechanism behind the light-induced FiM state and the presence of uncompensated magnetic moments in AFM MnPS_3_. To understand these phenomena, we calculated the time-dependent occupation (*n*(*t*) – *n*(0)) and time-dependent density of states (TDDOS) for Mn1 and Mn2 atoms, as depicted in Figure 3. Within the time window of [7, 15] fs after the peak of the laser pulse, we observed a loss/gain of majority (N↑) and minority (N↓) electrons in Mn1 and Mn2 atoms, leading to the demagnetization of Mn1 and Mn2. This demagnetization arises due to the magnetic moment being directly proportional to the difference between majority and minority electrons, that is, M ∝ (N↑ - N↓). Following the pulse peak (around ∼12 fs), the occupation reaches saturation, showing only minor modifications. Notably, the majority electron of Mn1 gradually decreases, while the majority electron of Mn2 shows a gradual increase. Surprisingly, the minority electrons of both Mn1 and Mn2 do not exhibit significant changes. This spin-dependent charge transfer leads to the presence of uncompensated magnetic moments and net magnetism in the AFM MnPS_3_. Additionally, the TDDOS reveals the existence of pronounced photoexcited majority states within the energy range of [1, 3] eV, corresponding to the occupation dynamics of the majority states of Mn1 and Mn2 atoms. Previous studies on metal multilayers and 2D magnetic heterostructures have revealed a close relationship between spin transfer and the occupation of unoccupied states located above the Fermi energy level. In the case of the Mn1 atom, the energy band structure displayed in Figure S1 indicates that the unoccupied states primarily originate from the spin-down state. Conversely, for the Mn2 atom, the unoccupied states arise from the spin-up states due to the spin antiparallel arrangement between Mn1 and Mn2 atoms. Consequently, the disparity in the empty states above the Fermi level induces distinct changes in the magnetic moments of Mn1 and Mn2, resulting in the emergence of a net magnetic moment and the establishment of the FiM state.

Spin transfer dynamics is usually dependent on the Fluence of the laser pulse. The dynamics of the magnetic moments of Mn1/Mn2 and the net magnetic moment in AFM MnPS_3_ under laser pulses with fluences of 24.6 mJ/cm^2^, 55.3 mJ/cm^2^, and 84.1 mJ/cm^2^ are illustrated in Figure S2 in the Supporting Information. Our findings reveal that increasing the fluence of the laser pulse intensifies the demagnetization behavior of Mn1 and Mn2 atoms. Interestingly, the dynamics of the net magnetic moment do not exhibit significant changes with increasing fluence. This can be attributed to the fact that despite enhanced demagnetization, the magnetic difference between Mn1 and Mn2 atoms does not substantially increase. However, it is worth noting that at higher fluences, the magnetic atoms may undergo a change in the direction of their magnetic moments. In fact, when the laser energy reaches 84.1 mJ/cm^2^, we observe the Mn1 atom undergoing a spin direction change around 35 fs, leading to a transition of magnetic order of MnPS_3_ from the FiM state to the FM state. After the magnetic order transition takes place, we also observe a significant alteration in the interlayer spin transfer between the Mn1 and Mn2 atoms. As a result, there is a pronounced decrease in the net magnetic moment occurring after 35 fs. Nevertheless, it should be noted that employing such high fluences (∼84.1 mJ/cm^2^) for optical manipulation of spin dynamics in 2D magnets may result in light-induced damage to the materials, rendering it unreliable for practical applications. We also investigated the impact of spin–orbit coupling (SOC) on the demagnetization dynamics of Mn1 and Mn2 atoms as well as the net magnetism, as illustrated in Figure S3. In the presence of SOC, we observed a persistent slow demagnetization of Mn1 and Mn2 beyond 30 fs, attributed to the occurrence of spin-flip processes. The introduction of SOC breaks the conservation of total spin moment as a good quantum number, thereby allowing for photoinduced spin-flip processes.

## Spin Relaxation Dynamics after Laser Pulse

**Figure 4:**
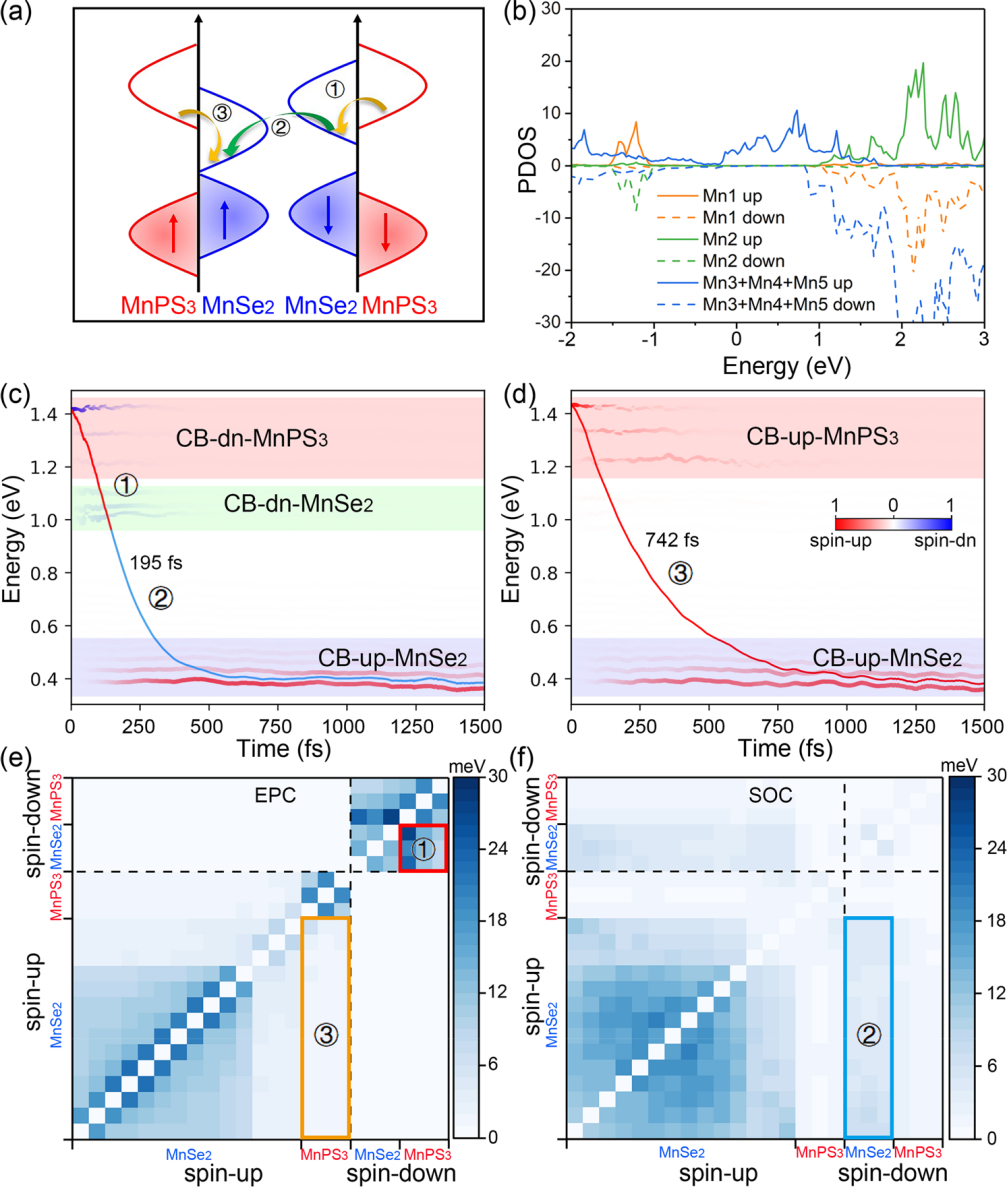
Spin electron relaxation dynamics of MnPS_3_/MnSe_2_heterostructure from NAMD simulations. (a) Schematic diagram of spin electron relaxation pathways. Yellow and green arrows represent the interlayer charge transfer and intralayer SF respectively. (b) PDOS of Mn atoms in the MnPS_3_ and MnSe_2_ layer. Mn1 and Mn2 originate from the MnPS_3_ layer, and Mn3, Mn4 and Mn5 originate from the MnSe_2_ layer. (c, d) Energy relaxation of the excited spin electron from the spin-up and spin-down states of MnPS_3_ respectively. Red and blue decay curves represent process ① and process ② respectively. Spin-dn CB of MnPS_3_, spin-dn CB of MnSe_2_ and spin-up CB of MnSe_2_ are marked as red, green, and blue regions, respectively, in Figure 4c. Spin-up CB of MnPS_3_ and MnSe_2_ are marked as red and blue regions, respectively, in Figure 4d. The color bar indicates the orbital localization. Process ①, process ③, and process ② mean the interlayer charge transfer between two layers and intralayer SF in the MnSe_2_ layer, respectively. The time data are fitted as a Gaussian function. (e, f) EPC and SOC components between key electronic states in the MnPS_3_/MnSe_2_ heterostructure according to the spin-diabatic representation. Red, blue, and yellow frames represent the corresponding NAC component of process ①, process ②, and process ③.

So far, we have demonstrated the emergence of a ferrimagnetic state in the AFM MnPS_3_ layer under excitation by the laser pulse. Upon the disappearance of the pulse, the excited spin electrons relax from higher energy levels to lower energy levels, potentially affecting the magnetism of the AFM Mn atoms, as shown in Figure 4a. To evaluate the change in the magnetic state of Mn atoms in such dynamic processes, we first investigate the partial density of states (PDOS) of Mn atoms in the MnPS_3_ and MnSe_2_ layers, as shown in Figure 4b. It is found that the spin-up and spin-down states in the conduction band (CB) of the MnPS_3_ layer are contributed by Mn2 and Mn1 atoms, respectively. As a result, the spin-up electrons from Mn2 atoms and spin-down electrons from Mn1 atoms transfer to the adjacent MnSe_2_ layer during the relaxation process. We further calculate the energy relaxation of spin-up and spin-down electrons based on spin-adiabatic representation by using *ab initio* NAMD simulations (Figure 4c and d). It is observed that the spin-up electrons of the Mn2 atoms directly transfer to the MnSe_2_ layer within 742 fs, which is fitted by a Gaussian function (Figure 4d). However, the relaxation process for spin-down electrons of Mn1 involves two stages: interlayer charge transfer (CT) (process ①) and intralayer spin-flip (SF) (process ②). The spin-down electrons of Mn1 atoms first transfer to the MnSe_2_ layer and then undergo SF from spin-down to spin-up states within 195 fs. Note that the faster interlayer CT between spin-down states of the two layers leads to a faster increase in the magnetic moment of Mn1 atoms compared to that of Mn2 atoms. As a result, the Mn1 and Mn2 atoms, accompanied by their inequivalent magnetic moment recovery, will stay in the FiM state on a subpicosecond time scale before reverting to the AFM state. Furthermore, the intralayer SF in the MnSe_2_ layer following the interlayer CT between two layers will also increase the magnetic moment of Mn atoms in the MnSe_2_ layer.

Based on the above results obtained from the NAMD simulations, we can provide a comprehensive description of the spin electron relaxation dynamics. Initially, the excited spin-up (spin-down) electrons from Mn2 (Mn1) atoms in the MnPS_3_ layer transfer to MnSe_2_, resulting in a decrease (increase) in the ratio of spin-up to spin-down electrons for Mn2 (Mn1) atoms. Consequently, the magnetic moment of Mn1 and Mn2 atoms increases, leading to the restoration of their magnetic order from the FiM state to the AFM state ultimately. It should be noted that these processes exhibit inequivalent transfer rates, causing the Mn1 and Mn2 atoms to maintain a FiM state within a longer subpicosecond time scale during the relaxation process. Additionally, the magnetic moment of Mn atoms in the MnSe_2_ layer also recovers due to the intralayer SF processes. In summary, the ferrimagnetic state in the MnPS_3_ layer gradually diminishes, transitioning back to an antiferromagnetic state, and the magnetic moment of Mn atoms in the MnSe_2_ layer is also restored.

To gain deeper insight into the factors influencing the spin relaxation dynamics, we conducted calculations of the nonadiabatic coupling (NAC) matrix between key electronic states in the MnPS_3_/MnSe_2_ heterostructure. The NAC matrix, as shown in Figure 4e and f, was determined using the spin-diabatic representation, which allows one to clearly distinguish the contributions from electron–phonon coupling (EPC) and SOC components. This distinction is essential for understanding the physical picture of spin dynamics.^32,33^ Specifically, the EPC component primarily governs the coupling between adjacent orbitals with the same spin states, whereas the SOC component encompasses not only supplies the coupling between identical spin states but also contributes to the coupling between opposite spin states. Our results reveal that the EPC component between the spin-down states of the two layers is significantly larger than that between the spin-up states, revealing the faster charge transfer rate associated with process ① in comparison to process ③. Furthermore, the SOC component between the spin-up and spin-down states in MnSe_2_ surpasses the EPC component between the spin-up states of the two layers. These results further indicate the increase in the net magnetism (uncompensated Mn magnetic moment) of MnPS_3_ before its recovery to an AFM state. To further estimate the effect of multielectron interaction on spin electron relaxation, the multielectron NAMD simulations are also performed in this work (see Figure S4). It can be seen that the energy relaxation time calculated by multielectron simulation is similar to that calculated by single electron simulation with a small difference in relaxation time but the same relaxation pathway (Figure 4c and d), indicating that our conclusion is still reasonable based on the multielectron NAMD simulation.

In conclusion, the rt-TDDFT simulations generally are limited to describing spin dynamics occurring on time scales shorter than 100 fs (OISTR effect). Beyond 100 fs, phonon-mediated spin relaxation processes significantly influence the magnetization dynamics of materials. Unfortunately, these processes have not been extensively explored by using quantum mechanical simulations. To address this gap, our study employed rt-TDDFT and ab initio NAMD to investigate the interlayer spin transfer and relaxation dynamics in 2D AFM/FM vdW heterostructures. Our findings demonstrate that laser pulses induce spin-selective charge transfer, resulting in the emergence of a FiM state and net magnetism within the AFM layer within tens of femtoseconds. The presence of uncompensated magnetic moments in the AFM layer is attributed to the distinct electronic structures and interlayer spin transfer of the sublattice in the AFM layer. Furthermore, we observed that the excited electrons of Mn1 and Mn2 exhibit inequivalent relaxation times of 195 and 742 fs, respectively, during the spin relaxation process. The faster interlayer charge relaxation leads to a more rapid increase in the magnetic moment of Mn1 atoms, revealing the sustained FiM state on the subpicosecond time scale. This transition from AFM to FiM state and the persistence of subpicosecond FiM states are attributed to the asymmetrical feature of the interlayer OISTR effect and the unequal electron–phonon interaction of spin-up and spin-down channels of the AFM spin sublattice. These results present a promising pathway toward optically manipulating interlayer spin transfer and relaxation dynamics in vdW magnetic heterostructures.

## Supporting Information Available

Computational methods, Mn atoms projected band structures, fluence-dependent magnetization dynamics, magnetization dynamics with and without spin–orbital coupling, and multielectron NAMD simulations (PDF)

## References

[ref1] Kirilyuk A.; Kimel A. V.; Rasing T. Ultrafast optical manipulation of magnetic order. Rev. Mod. Phys. 2010, 82, 2731 10.1103/RevModPhys.82.2731.

[ref2] Baltz V.; et al. Antiferromagnetic spintronics. Rev. Mod. Phys. 2018, 90, 015005 10.1103/RevModPhys.90.015005.

[ref3] Jungwirth T.; Marti X.; Wadley P.; Wunderlich J. Antiferromagnetic spintronics. Nat. Nanotechnol. 2016, 11, 231–241. 10.1038/nnano.2016.18. 26936817

[ref4] Thielemann-Kühn N.; et al. Ultrafast and energy-efficient quenching of spin order: antiferromagnetism beats ferromagnetism. Phys. Rev. Lett. 2017, 119, 197202 10.1103/PhysRevLett.119.197202. 29219516

[ref5] Ju G.; et al. Ultrafast generation of ferromagnetic order via a laser-induced phase transformation in FeRh thin films. Phys. Rev. Lett. 2004, 93, 197403 10.1103/PhysRevLett.93.197403. 15600878

[ref6] Thiele J. U.; Buess M.; Back C. H. Spin dynamics of the antiferromagnetic-to-ferromagnetic phase transition in FeRh on a sub-picosecond time scale. Appl. Phys. Lett. 2004, 85, 2857–2859. 10.1063/1.1799244.

[ref7] Golias E. I.; et al. Ultrafast optically induced ferromagnetic state in an elemental antiferromagnet. Phys. Rev. Lett. 2021, 126, 107202 10.1103/PhysRevLett.126.107202. 33784145

[ref8] Lee J. U.; et al. Ising-type magnetic ordering in atomically thin FePS_3_. Nano Lett. 2016, 16, 7433–7438. 10.1021/acs.nanolett.6b03052. 27960508

[ref9] Kim K.; Lim S. Y.; Lee J.-U.; Lee S.; Kim T. Y.; Park K.; Jeon G. S.; Park C.-H.; Park J.-G.; Cheong H.; et al. Suppression of magnetic ordering in XXZ-type antiferromagnetic monolayer NiPS_3_. Nat. Commun. 2019, 10, 345 10.1038/s41467-018-08284-6. 30664705 PMC6341093

[ref10] Long G.; et al. Isolation and characterization of few-layer manganese thiophosphite. ACS Nano 2017, 11, 11330–11336. 10.1021/acsnano.7b05856. 29023097

[ref11] Deng Y.; et al. Gate tunable room-temperature ferromagnetism in two-dimensional Fe_3_GeTe_2_. Nature 2018, 563, 94–99. 10.1038/s41586-018-0626-9. 30349002

[ref12] Huang B.; et al. Layer-dependent ferromagnetism in a van der Waals crystal down to the monolayer limit. Nature 2017, 546, 270–273. 10.1038/nature22391. 28593970

[ref13] Gong C.; et al. Discovery of intrinsic ferromagnetism in two-dimensional van der Waals crystals. Nature 2017, 546, 265–269. 10.1038/nature22060. 28445468

[ref14] Li D.; Li S.; Zhong C.; He J. Tuning magnetism at the two-dimensional limit: A theoretical perspective. Nanoscale 2021, 13, 19812–19827. 10.1039/D1NR06835K. 34825688

[ref15] Li D.; Haldar S.; Heinze S. Strain-Driven Zero-Field Near-10 nm Skyrmions in Two-Dimensional van der Waals Heterostructures. Nano Lett. 2022, 22, 7706–7713. 10.1021/acs.nanolett.2c03287. 36121771

[ref16] Zhong D.; Seyler K. L.; Linpeng X.; Cheng R.; Sivadas N.; Huang B.; Schmidgall E.; Taniguchi T.; Watanabe K.; McGuire M. A.; et al. Van der Waals engineering of ferromagnetic semiconductor heterostructures for spin and valleytronics. Sci. Adv. 2017, 3, e1603113 10.1126/sciadv.1603113. 28580423 PMC5451195

[ref17] Zhang P.; et al. All-optical switching of magnetization in atomically thin CrI3. Nat. Mater. 2022, 21, 1373–1378. 10.1038/s41563-022-01354-7. 36109674

[ref18] Liu B.; Liu S.; Yang L.; Chen Z.; Zhang E.; Li Z.; Wu J.; Ruan X.; Xiu F.; Liu W.; et al. Light-tunable ferromagnetism in atomically thin Fe3GeTe2 driven by femtosecond laser pulse. Phys. Rev. Lett. 2020, 125, 267205 10.1103/PhysRevLett.125.267205. 33449751

[ref19] Padmanabhan P.; Buessen F. L.; Tutchton R.; Kwock K. W. C.; Gilinsky S.; Lee M. C.; McGuire M. A.; Singamaneni S. R.; Yarotski D. A.; Paramekanti A.; et al. Coherent helicity-dependent spin-phonon oscillations in the ferromagnetic van der Waals crystal CrI3. Nat. Commun. 2022, 13, 4473 10.1038/s41467-022-31786-3. 35918314 PMC9345964

[ref20] Mertens F.; Monkebuscher D.; Parlak U.; Boix-Constant C.; Manas-Valero S.; Matzer M.; Adhikari R.; Bonanni A.; Coronado E.; Kalashnikova A. M.; et al. Ultrafast coherent THz lattice dynamics coupled to spins in the van der Waals antiferromagnet FePS3. Adv. Mater. 2023, 35, 2208355 10.1002/adma.202208355. PMC11475339 36437480

[ref21] Khela M.; Dabrowski M.; Khan S.; Keatley P. S.; Verzhbitskiy I.; Eda G.; Hicken R. J.; Kurebayashi H.; Santos E. J. G.; et al. Laser-induced topological spin switching in a 2D van der Waals magnet. Nat. Commun. 2023, 14, 1378 10.1038/s41467-023-37082-y. 36914683 PMC10011585

[ref22] Seyler K. L.; et al. Valley manipulation by optically tuning the magnetic proximity effect in WSe2/CrI3 heterostructures. Nano Lett. 2018, 18, 3823–3828. 10.1021/acs.nanolett.8b01105. 29756784

[ref23] Dewhurst J. K.; Elliott P.; Shallcross S.; Gross E. K.; Sharma S. Laser-induced intersite spin transfer. Nano Lett. 2018, 18, 1842–1848. 10.1021/acs.nanolett.7b05118. 29424230

[ref24] Elliott P.; Muller T.; Dewhurst J. K.; Sharma S.; Gross E. K. U.; et al. Ultrafast laser induced local magnetization dynamics in Heusler compounds. Sci. Rep. 2016, 6, 38911 10.1038/srep38911. 27966585 PMC5155284

[ref25] Willems F.; von Korff Schmising C.; Struber C.; Schick D.; Engel D. W.; Dewhurst J. K.; Elliott P.; Sharma S.; Eisebitt S.; et al. Optical inter-site spin transfer probed by energy and spin-resolved transient absorption spectroscopy. Nature Communi. 2020, 11, 1–7. 10.1038/s41467-020-14691-5. PMC7018696 32054855

[ref26] Hofherr M.; Hauser S.; Dewhurst J. K.; Tengdin P.; Sakshath S.; Nembach H. T.; Weber S. T.; Shaw J. M.; Silva T. J.; Kapteyn H. C.; et al. Ultrafast optically induced spin transfer in ferromagnetic alloys. Sci. Adv. 2020, 6, eaay8717 10.1126/sciadv.aay8717. 32010774 PMC6968944

[ref27] Tengdin P.; Gentry C.; Blonsky A.; Zusin D.; Gerrity M.; Hellbruck L.; Hofherr M.; Shaw J.; Kvashnin Y.; Delczeg-Czirjak E. K.; et al. Direct light–induced spin transfer between different elements in a spintronic Heusler material via femtosecond laser excitation. Sci. Adv. 2020, 6, eaaz1100 10.1126/sciadv.aaz1100. 32010777 PMC6968936

[ref28] Siegrist F.; et al. Light-wave dynamic control of magnetism. Nature 2019, 571, 240–244. 10.1038/s41586-019-1333-x. 31243366

[ref29] Steil D.; et al. Efficiency of ultrafast optically induced spin transfer in Heusler compounds. Phys. Rev. Res. 2020, 2, 023199 10.1103/PhysRevResearch.2.023199.

[ref30] Jiang M.; Xu K.; Liao N.; Zhou H. DFT investigation on highly selective NO2 sensing properties of MnPS_3_. Appl. Surf. Sci. 2021, 543, 148846 10.1016/j.apsusc.2020.148846.

[ref31] Kan M.; Adhikari S.; Sun Q. Ferromagnetism in MnX2 (X= S, Se) monolayers. Phys. Chem. Chem. Phys. 2014, 16, 4990–4994. 10.1039/c3cp55146f. 24477572

[ref32] Zheng Z.; Zheng Q.; Zhao J. Spin-orbit coupling induced demagnetization in Ni: Ab initio nonadiabatic molecular dynamics perspective. Phys. Rev. B 2022, 105, 085142 10.1103/PhysRevB.105.085142.

[ref33] Lei Y.; et al. Enhanced Electron Transfer and Spin Flip through Spin-Orbital Couplings in Organic/Inorganic Heterojunctions: A Nonadiabatic Surface Hopping Simulation. J. Phys. Chem. Lett. 2022, 13, 4840–4848. 10.1021/acs.jpclett.2c01177. 35616399

