# Supplementary material for: Ultrafast Laser Pulse Induced Transient Ferrimagnetic State and Spin Relaxation Dynamics in Two-Dimensional Antiferromagnets
Source: Nano Lett. 2023 Aug 29;23(17):8348–54. doi: 10.1021/acs.nanolett.3c02727 (PMC10510573; doi:10.1021/acs.nanolett.3c02727)
Supplement: Supplementary file 1 — nl3c02727_si_001.pdf [file nl3c02727_si_001.pdf]

Supplementary Materials "**Ultrafast laser pulse induced transient ferrimagnetic state and spin relaxation dynamics in two-dimensional antiferromagnets**"

Junjie He[1*], Shuo Li[2], Thomas Frauenheim[3], Zhaobo Zhou[4*]

[1] *Faculty of Science, Department of Physical and Macromolecular Chemistry, Charles University, Prague 12843, Czech Republic*

[2]*Institute of Advanced Study, Chengdu University, Chengdu 610100, China*

[3]*School of Science, Constructor University, Bremen 28759, Germany*

[4]*Bremen Center for Computational Materials Science, University of Bremen, Bremen 28359, Germany*

Corresponding Author:

E-mail: junjie.he.phy@gmail.com

E-mail: zzhou@uni-bremen.de

Table of Contents:

Computational methods, page S2-3

Mn atoms projected band structures, page S4

Fluence dependent magnetization dynamics, page S5

Magnetization dynamics with and without spin-orbital coupling, page S6

Multielectron NAMD simulations, page S7

Reference, page S8

Computational Methods

All the ground state calculations were implemented with the Vienna Ab initio Simulation Package (VASP).[1,2] The exchange-correlation interaction was treated with the Perdew-Burke-Ernzerhof (PBE) functional in the framework of the generalized gradient approximation.[3] The projector-augmented wave method was adopted to describe the electron-ion interaction.[4] An energy cutoff of 500 eV and a Monkhorst-Pack 7×7×1 k-mesh grid were adopted for geometry optimization and electronic structure calculations. The lattice constants and atomic positions were fully relaxed until the atomic forces were smaller than 0.01 eV $Å^{-1}$. The electron relaxation convergence criterion was 10^{-5} eV. To describe the Mn-d orbital better, the effective Hubbard U was set as 3 eV.[5] The van der Waals weak interaction was considered using the Grimme DFT-D3. To avoid the interaction between two neighboring periodic units, a vacuum region of 15 Å along the out-of-plane direction was used.

To explore the laser pulse induced spin dynamics, we performed rt-TDDFT calculations. The time evolving state functions (ψ) were calculated by solving the time dependent Kohn−Sham (KS) equation as follows:

$$i\frac{\partial\psi_j(\mathbf{r},t)}{\partial t} = \left[\frac{1}{2}\left(-i\nabla + \frac{1}{c}\mathbf{A_{ext}}(t)\right)^2 + v_s(\mathbf{r},t) + \frac{1}{2c}\sigma\cdot\mathbf{B_s}(\mathbf{r},t) + \frac{1}{4c^2}\sigma\cdot\left(\nabla v_s(\mathbf{r},t)\times -i\nabla\right)\right]\psi_j(\mathbf{r},t) \quad (1)$$

where $\mathbf{A_{ext}}(t)$ and σ represent vector potential and Pauli matrices, respectively. The KS effective potential $v_s(\mathbf{r},t) = v_{ext}(\mathbf{r},t) + v_H(\mathbf{r},t) + v_{xc}(\mathbf{r},t)$ contained three term: external potential v_{ext}, the classical Hartree potential v_H, and the exchange-correlation (XC) potential v_{xc}. The KS magnetic field can be written as $\mathbf{B_s}(\mathbf{r},t) = \mathbf{B_{ext}}(\mathbf{r},t) + \mathbf{B_{xc}}(\mathbf{r},t)$, where $\mathbf{B_{ext}}$ and $\mathbf{B_{xc}}$ represent the magnetic field of the applied laser pulse plus an additional magnetic field and XC magnetic field, respectively. The last term in Eq. (1) stands for SOC term. During the rt-TDDFT simulations, we froze the motion of nuclei all the time.

The rt-TDDFT simulations of MnPS3/MnSe2 heterostructures were made by using a fully non-collinear version of rt-TDDFT and a full-potential augmented plane-wave ELK code.[6] We used a regular mesh in a k-space of 5 × 5 × 1 with a smearing width of 0.027 eV for calculation of spin dynamics. The time step was set for $\Delta t = 0.1$ a.u. The laser pulses that were used in the present study were linearly polarized (in-plane polarization) at a selected frequency. All calculations were performed using adiabatic local spin density approximations (ALSDA) [7] with Hubbard U (U = 3 eV) for Mn atoms.

The spin electron relaxation dynamics simulations were implemented with the Hefei-NAMD code (Hefei-NAMD_SOC version).[8,9] After the geometry structures were optimized using VASP, the relaxed structures were warmed up to 300 K during 2 ps through repeated velocity rescaling. Then a 3 ps ab initio molecular dynamics (AIMD) trajectory was generated in the microcanonical ensemble with a time step of 1 fs. 200 initial configurations were selected randomly in the first 1 ps AIMD trajectory, and 20000 NAMD trajectories were sampled for each chosen initial structure.

Mn atoms projected band structures.

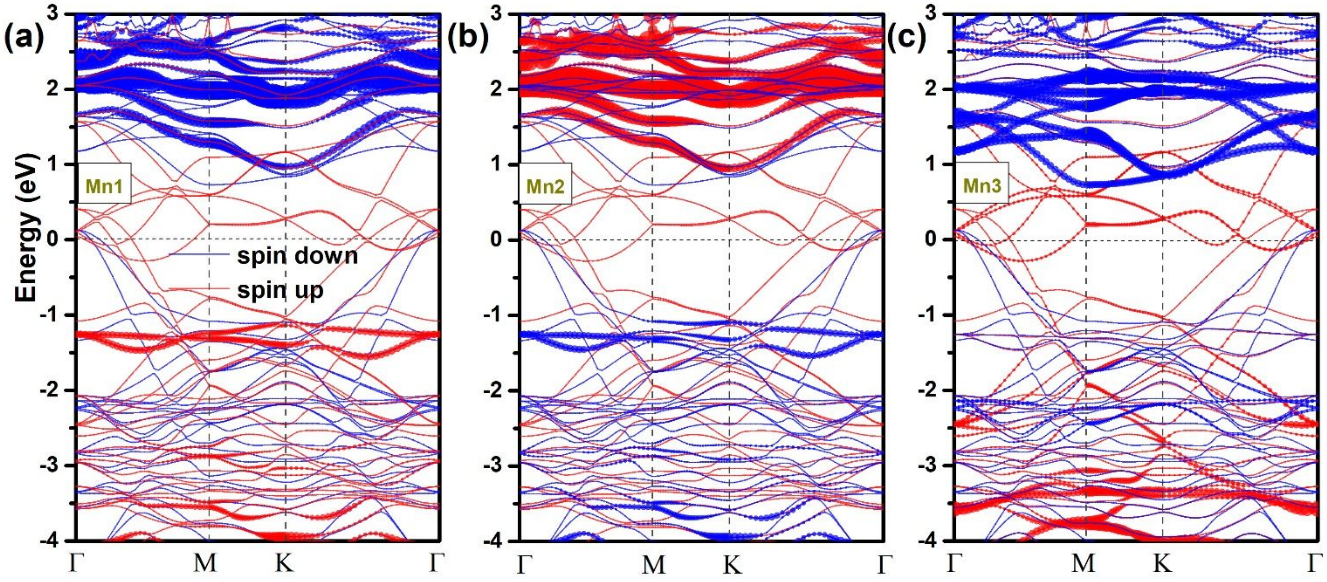

Figure S1. Atom-projected band structures. Band structures of (a) Mn1, (b) Mn2 and (c) Mn3, respectively. Red and blue represent the spin up and spin down, respectively.

Fluence dependent magnetization dynamics

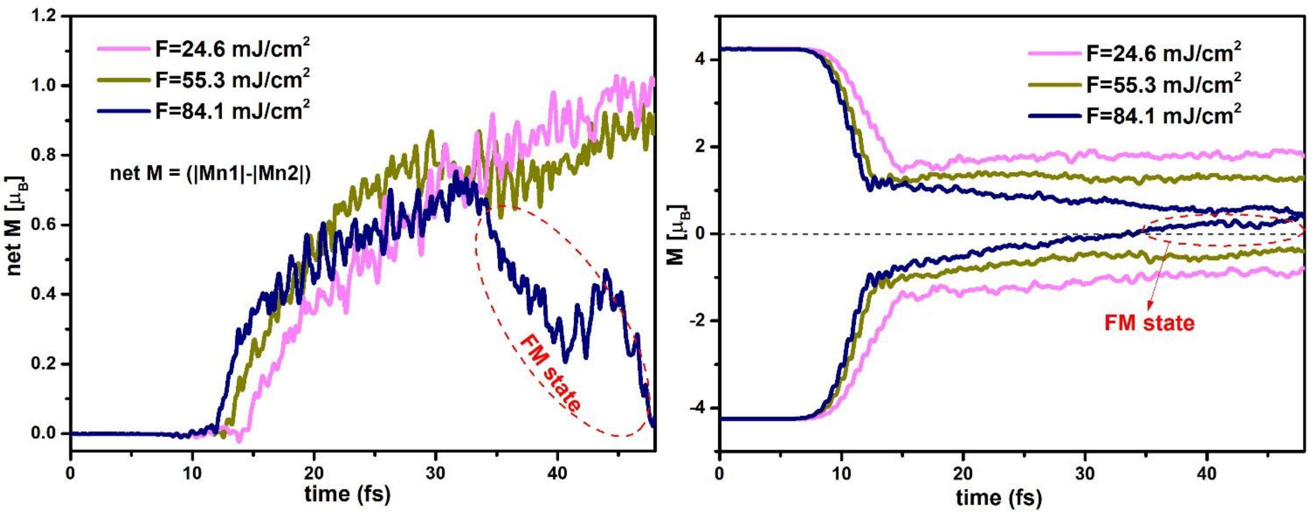

Figure S2. The laser Fluence dependent spin dynamics. Laser pulse-induced dynamics of **(a)** net magnetic moment and **(b)** local magnetic moment of Mn1/Mn2 atom at different laser fluences (F) of 24.6 mJ/cm^2, 55.3 mJ/cm^2, and 84.1 mJ/cm^2. Notable ferromagnetic (FM) state transition under high-energy laser power is highlighted.

Magnetization dynamics with and without spin-orbital coupling

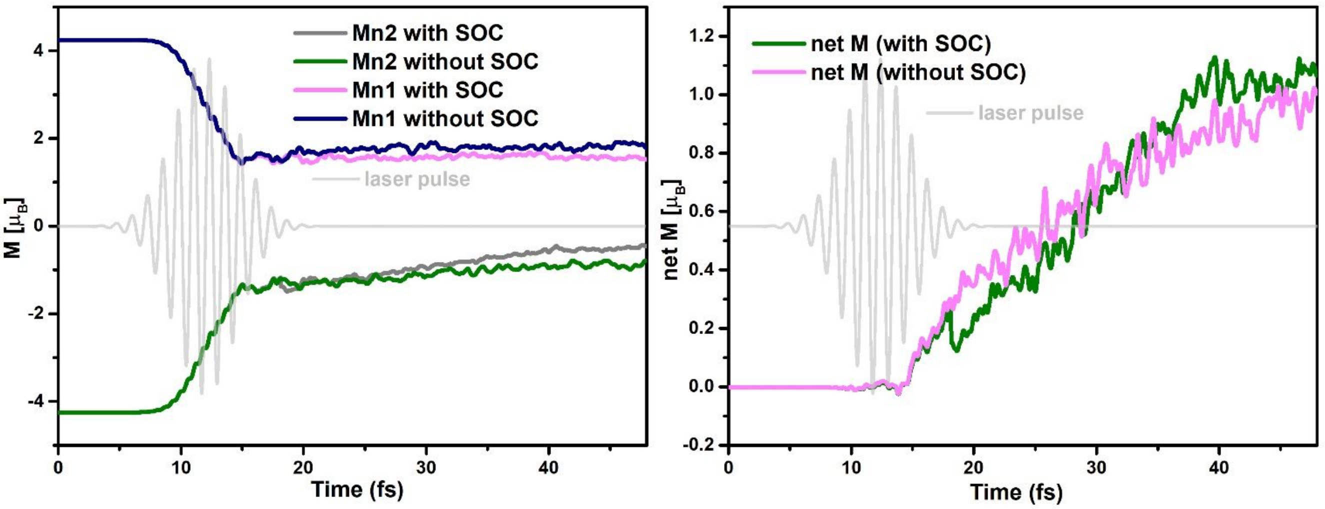

Figure S3. Spin dynamics with and without SOC. Dynamics of (a) local magnetic moment**,** Mn1/Mn2 and (b) net magnetic moment as a function of time (in fs) with and without spin-orbit coupling (SOC).

Multielectron NAMD simulations

More than one electron is excited to a series of electronic states, and the Pauli repulsion principle is considered when simulating their relaxation. Here, the number of excited spin-up electrons in the $MnPS_3$ layer is set to be one more than the number of spin-down electrons due to the emergence of a net magnetic moment after laser excitation (See Fig. 2a). The energy relaxation of the excited spin electrons from the spin-up and spin-down states of $MnPS_3$ using multi-electron NAMD simulation is shown in Fig. S4.

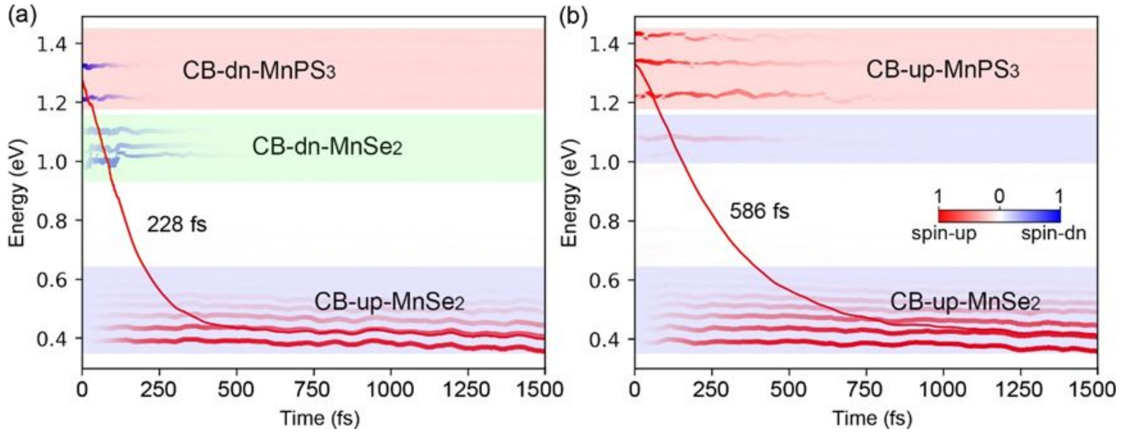

Figure S4. Energy relaxation of excited spin electrons from the spin-up and spin-down states of $MnPS_3$ using multi-electron NAMD simulation. Spin-dn CB of $MnPS_3$, spin-dn CB of $MnSe_2$ and spin-up CB of $MnSe_2$ are marked as red, green and blue regions respectively in Fig. S4a. Spin-up CB of $MnPS_3$ and $MnSe_2$ are marked as red and blue regions respectively in Fig. S4b. The time data is fitted as a Gaussian function.

Reference:

1. Kresse, G.; Furthmuller, J. Efficient iterative schemes for ab initio total-energy calculations using a plane-wave basis set. *Phys. Rev. B: Condens. Matter Mater. Phys.* **1966**, *54*, 11169-11186.

2. Kresse, G.; Hafner, J. Ab initio molecular-dynamics simulation of the liquid-metal-amorphous-semiconductor transition in germanium. *Phys. Rev. B: Condens. Matter Mater. Phys.* **1944**, *49*, 14251-14269.

3. Perdew, J. P.; Burke, K.; Ernzerhof, M. Generalized gradient approximation made simple. *Phys. Rev. Lett.* **1966**, *77*, 3865-3868.

4. Kresse, G.; Joubert, D. From ultrasoft pseudopotentials to the projector augmented-wave method. *Phys. Rev. B: Condens. Matter Mater. Phys.* **1999**, *59*, 1758-1775.

5. He, J. et al. Ultrafast Light-Induced Ferromagnetic State in Transition Metal Dichalcogenides Monolayers. *J. Phys. Chem. Lett.* **2022**, *13*, 2765-2771.

6. Dewhurst, J. K.; Sharma, S. et al. Elk code, elk.sourceforge.net (accessed Oct 1, 2020).

7. von Barth, U.; Hedin, L. A local exchange-correlation potential for the spin polarized case, *J. Phys. C: Solid State Phys.* **1972**, *5*, 1629.

8. Zheng, Z.; Zheng, Q.; Zhao, J. Spin-orbit coupling induced demagnetization in Ni: Ab initio nonadiabatic molecular dynamics perspective. Phys. Rev. B **2022**, *105*, 085142.

9. Zheng, Q. et al. Ab initio nonadiabatic molecular dynamics investigations on the excited carriers in condensed matter systems. *WIREs Comput Mol Sci.* **2019**, *9*, e1411.
